# Supplementary material for: Variation in multiple classes of simple sequence repeats can alter drug susceptibility in Mycobacterium tuberculosis
Source: bioRxiv. 2026 Sep 19:2026.09.16.752248. Preprint. [Version 1] doi: 10.64898/2026.09.16.752248 (PMC13596474; doi:10.64898/2026.09.16.752248)
Supplement: Supplementary file 1 [file NIHPP2026.09.16.752248v1-supplement-1.pdf]

bioRxiv preprint doi: https://doi.org/10.64898/2026.09.16.752248; this version posted September 20, 2026. The copyright holder for this preprint (which was not certified by peer review) is the author/funder, who has granted bioRxiv a license to display the preprint in perpetuity. It is made available under a CC-BY 4.0 International license.

Supporting Information

Figure S1 – Distribution of *ponA1* proline repeats in L1, L2, and L4 of *M. tuberculosis.* **(A)** Schema of *ponA1* proline repeats (left) and phylogenetic distribution of the fixed CCG > TCG (Pro > Ser) across 4 *Mtb* lineages. **(B)** Phylogenetic relationship of proline repeats across L1, L2, and L4 of *Mtb*.

Table S1: Information on publicly available WGS of Mtb isolates used in this work

Table S2: Homoplasy scores of SSR variants and DR SNVs in Mtb isolates from Peru and Vietnam

Table S3: Data on the antibiotic resistance association test on Mtb isolates from Vietnam and Peru.

Table S4: Oligos and primers used in this study

Figure S1

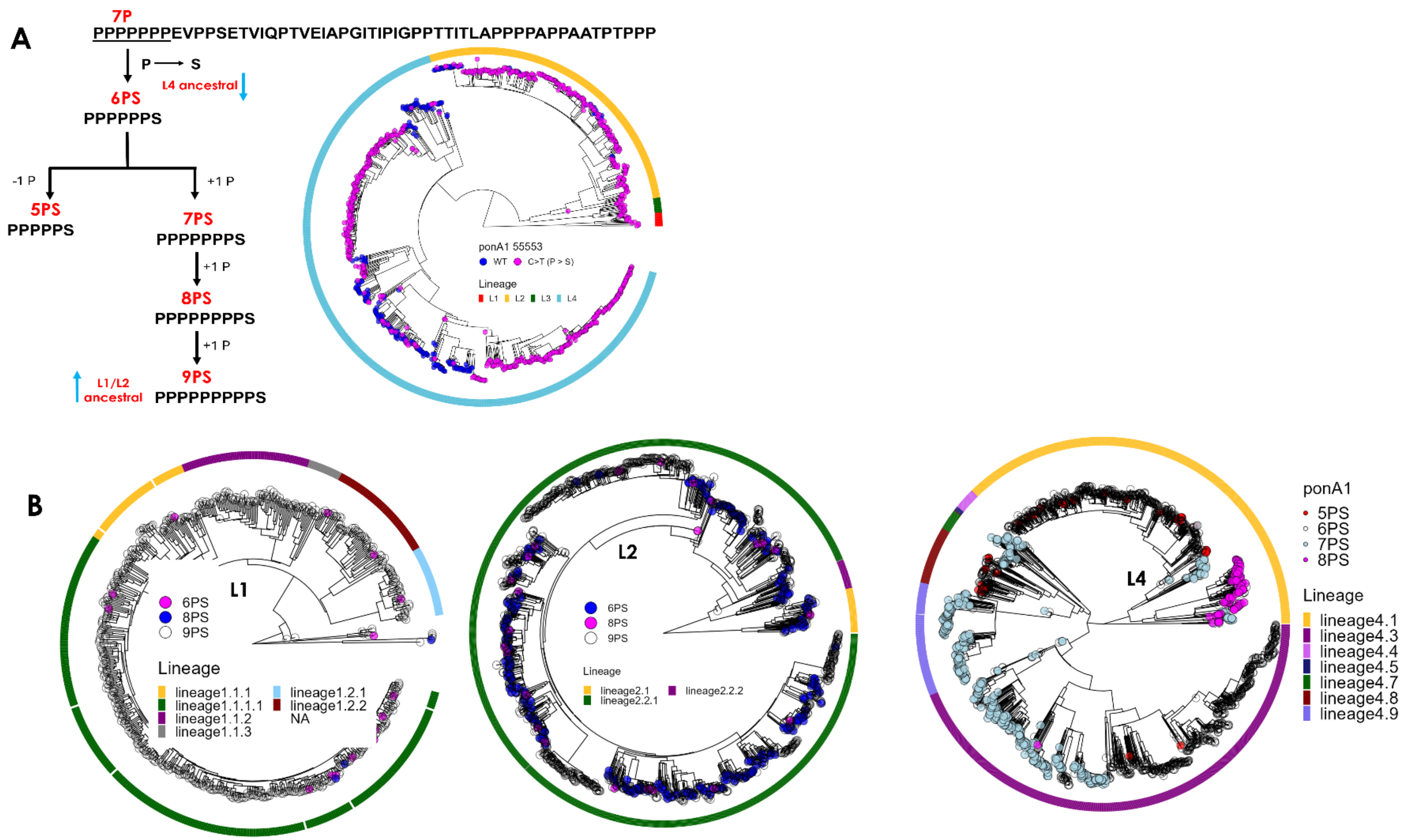
A
7P
PPPPPPPEVPPSETVIQPTVEIAPGITIPIGPPTTITLAPPPPAPPAATPTPPP
P → S
L4 ancestral
6PS
PPPPPPS
-1 P
+1 P
5PS
PPPPPS
7PS
PPPPPPPS
+1 P
8PS
PPPPPPPPS
+1 P
9PS
PPPPPPPPPS
L1/L2 ancestral
ponA1 55553
WT
C>T (P > S)
Lineage
L1
L2
L3
L4
B
L1
6PS
8PS
9PS
Lineage
lineage1.1.1
lineage1.1.1.1
lineage1.1.2
lineage1.1.3
lineage1.2.1
lineage1.2.2
NA
L2
6PS
8PS
9PS
Lineage
lineage2.1
lineage2.2.1
lineage2.2.2
L4
ponA1
5PS
6PS
7PS
8PS
Lineage
lineage4.1
lineage4.3
lineage4.4
lineage4.5
lineage4.7
lineage4.8
lineage4.9
